# Nacre-Inspired Flexible Mxene-Based Films for Multifunctional Applications in Supercapacitors and Piezoresistive Sensors

**DOI:** 10.3390/s26123762

**Published:** 2026-06-12

**Authors:** Beibei Wang, Licheng Zhou, Sentao Wei, Qiuhang Zhu, Qun Wu, Chuan Cao

**Affiliations:** Lab of Material Innovation Design and Intelligent Interaction, Zhejiang Sci-Tech University, Hangzhou 311199, China; ber@zstu.edu.cn (B.W.); 2025211205032@mails.zstu.edu.cn (L.Z.); 2024211205022@mails.zstu.edu.cn (S.W.); 2025211205034@mails.zstu.edu.cn (Q.Z.)

**Keywords:** MXene, cellulose nanofiber, multifunctional applications, piezoresistance sensor, supercapacitor

## Abstract

The explosive demand for flexible wearable and portable devices imposes stringent requirements on the mechanical, energy storage, and sensing properties of functional materials. Although two-dimensional (2D) transition metal carbides and nitrides (MXene) possess high conductivity and pseudocapacitance, their severe self-restacking and intrinsic brittleness restrict their practical applications. Herein, a facile vacuum filtration and hot-pressing densification strategy is proposed to fabricate nacre-inspired MXene-based films. By incorporating one-dimensional (1D) high-aspect-ratio TEMPO-oxidized cellulose nanofibrils (TOCNFs), the self-restacking of MXene is effectively suppressed. The optimal M20F5 composite film exhibits a coordinated electromechanical balance, maintaining an electrical conductivity of 1.07 × 10^6^ S m^−1^ while enduring 2124 folding cycles. For energy storage, the assembled symmetric supercapacitor delivers a specific capacitance of 828.92 F g^−1^ at 0.5 mA cm^−2^ and maintains an energy density of 13.75 Wh kg^−1^ at a power density of 9500 W kg^−1^. Furthermore, acting as a piezoresistive sensor, the film achieves reliable detection, spanning from bimodal gait recognition to subtle physiological pulses. This work establishes a viable material design strategy for next-generation supercapacitors and intelligent wearable systems.

## 1. Introduction

Recently, the surging interest in flexible wearable and portable devices, spanning personal health monitoring [1], human–machine interaction [2], and medical diagnostics [3], has triggered an explosive demand for advanced energy storage technologies and highly sensitive sensors [4]. Supercapacitors, owing to their high power density, rapid charge–discharge capability, and outstanding cycling stability, represent ideal energy storage candidates to satisfy the instantaneous high-power demands and prolong the operational lifespan of portable electronics [5]. Concurrently, piezoresistive sensors play a vital role in signal monitoring scenarios due to their high sensitivity, rapid response, and structural simplicity [6]. Since material properties directly dictate the overall device performance, developing versatile functional materials that integrate flexibility, mechanical robustness [7], and high electrical conductivity has emerged as a critical research imperative across materials science and electronic engineering.

MXene have attracted widespread attention in academia owing to their high electrical conductivity and abundant surface functional groups [8,9]. Benefiting from their excellent intrinsic conductivity and 2D layered structure, MXene can construct conductive networks that are highly sensitive to minute deformations and yield rapid signal responses [10,11]. The rich surface functional groups of MXene provide a vast number of electrochemical active sites and outstanding pseudocapacitive characteristics for energy storage [12]. However, during film fabrication, MXene nanosheets suffer from severe self-restacking driven by strong van der Waals forces and hydrogen bonding, rendering them highly susceptible to brittle fracture [13,14,15]. Consequently, pure MXene films fail to satisfy the stringent durability standards required for supercapacitors and piezoresistive sensors. To suppress this self-restacking phenomenon, researchers have explored various material design strategies based on micro-nano structures, among which introducing low-dimensional nanomaterials as physical spacers has been demonstrated as an effective approach [16]. To address this challenge, 1D high-aspect-ratio TOCNFs are introduced to intercalate between the MXene layers, effectively alleviating the self-restacking of MXene nanosheets [17,18]. The abundant oxygen-containing functional groups on the TOCNFs surface impart excellent water dispersibility, enabling homogeneous blending with hydrophilic MXene in the aqueous phase [19]. Furthermore, TOCNFs can form a high-density interfacial hydrogen-bonding network with MXene, which effectively resolves the issue of weak interfacial adhesion and significantly enhances the mechanical properties of the composites [20].

Currently, the MXene and TOCNFs composite system exhibits a clear trend of synergistic development in both energy storage and sensor fields. Within the energy storage sector, Ahmad et al. demonstrated that TOCNFs can effectively serve as physical spacers, expanding the interlayer spacing while exposing more electrochemical active sites, thereby significantly enhancing ion accessibility and charge storage capacity [21]. Chen et al. reported that the abundant hydrophilic functional groups of TOCNFs efficiently improve the wettability between the electrode material and aqueous electrolytes, guaranteeing the device’s structural stability under high rate charge–discharge conditions [22]. In the sensor field, Han et al. indicated that TOCNFs, serving as a flexible binder integrated with MXene, enable the fabrication of films with a nacre-inspired lamellar network architecture, endowing them with excellent mechanical robustness to withstand dynamic mechanical fatigue. Additionally, the material can generate substantial resistance signal transduction through interlayer sliding or dynamic variation in interlayer spacing [23]. Although our research team [24] and other related studies [25,26,27,28,29,30] have sought to balance mechanical and electrochemical performance, they typically necessitate the introduction of a third component or rely on complex chemical cross-linking methods. Consequently, achieving a dual breakthrough in both energy storage activity and sensing sensitivity through a simpler, greener physical regulation strategy while maintaining system simplicity remains a critical frontier challenge in the field of flexible electronics.

**Figure 1:**
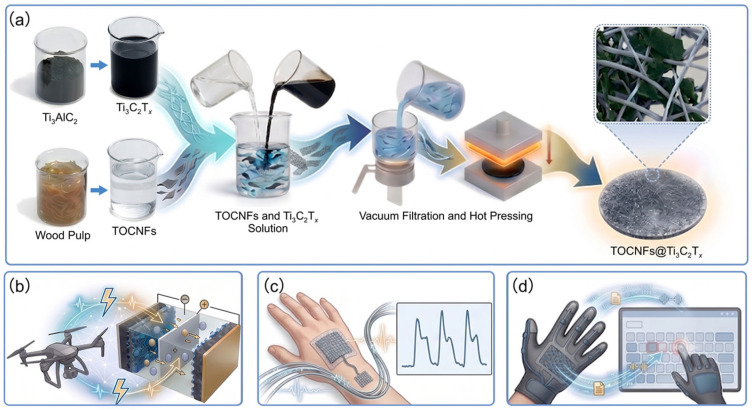
(**a**) Schematic illustration for preparing MXene-based films; (**b**–**d**) applications of MXene-based films.

Herein, a facile vacuum filtration and hot-pressing densification strategy is proposed (Figure 1a) to fabricate MXene-based films with a nacre-inspired lamellar network architecture, successfully integrating flexibility, robust mechanical properties, and high electrical conductivity. Unlike previous works relying on multi-component additives or intricate chemical cross-linking, this study demonstrates that MXene/TOCNFs network can achieve unprecedented synergistic performance solely via physical tailoring. The hot-pressing process eliminates microscopic voids, significantly reducing TOCNFs-induced interfacial contact resistance and facilitates a dense hydrogen-bonding network [8]. Acting as elastic nano-binders and physical spacers, the high-aspect-ratio TOCNFs effectively suppress MXene self-restacking, endowing the film with exceptional dynamic mechanical fatigue resistance and long-term operational stability. Under external mechanical stimuli, the microscopic constituent layers undergo controllable relative sliding to alter the contact area, inducing resistance variations for highly sensitive force-to-electricity signal transduction. Meanwhile, the remodeled microscopic pore architecture effectively suppresses the self-restacking phenomenon, exposing abundant pseudocapacitive active sites and ensuring highly accessible ion-transport pathways, which enables the device to achieve high specific capacitance and outstanding electrochemical cycling stability. By balancing accurate perception and highly efficient energy storage, the composite film presents a highly promising solution for next-generation high-performance supercapacitors, personal health monitoring, and flexible wearable devices (Figure 1b–d).

## 2. Materials and Methods

### 2.1. Materials

Sodium bromide (NaBr, 99%), sodium hypochlorite (NaClO, 99%) and sodium hydroxide (NaOH, 99%) were purchased from Sinopharm Chemical Reagent Co., Ltd. (Shanghai, China). The bleached softwood pulp was sourced from Donghua Pulp Factory, China. Titanium Aluminum Carbide (Ti_3_AlC_2_, MAX) powders were supplied by Hangzhou Nano-Mall Technology Co. Ltd. (Hangzhou, China). All reagents and chemicals were used as received without further purification.

### 2.2. Preparation of Ti_3_C_2_T_x_

Ti_3_C_2_T*_x_* was synthesized by etching the Ti_3_AlC_2_ MAX phase [31]. Stirred LiF (1.6 g) and HCl (20 mL, 9 M) solution for 5 min, Ti_3_AlC_2_ was added slowly (about 10 min) and stirred at 45 °C for 24 h. Then washing was repeated and it was centrifuged 7 times with DI water until the pH was ≥6. Finally, the product was delaminated by ice-bath sonication, and the target Ti_3_C_2_T*_x_* dispersion (5 mg mL^−1^) was isolated by a final centrifugation at 3500 rpm.

### 2.3. Preparation of TOCNFs

To prepare TOCNFs, 1g of softwood pulp was dispersed in deionized water (DI, 100 mL) along with TEMPO (0.016 g, 0.1 × 10^−3^ M) and NaBr (0.1 g, 1 × 10^−3^ M). The oxidation was activated by adding NaClO (5 mmol g^−1^), while a 0.5 M NaOH solution was utilized to stabilize the pH at 10.0–10.5. After a 5 h reaction, the mixture was quenched with ethanol and neutralized. The oxidized cellulose was washed, centrifuged, and homogenized to yield a 1 wt.% TOCNFs dispersion.

### 2.4. Preparation of MXene-Based Films

A combined vacuum filtration and hot-pressing strategy was employed to construct the MXene-based films. Dispersions of Ti_3_C_2_T*_x_* and TOCNFs were blended at specified weight proportions and diluted with DI water, yield a homogeneous suspension (20 mL) with a total concentration of 1.25 mg mL^−1^. Following ultrasonic homogenization, the mixture was deposited onto a porous membrane via vacuum filtration. To promote interfacial adhesion and eradicate microscopic voids within the structure, the as-filtered wet paper was sandwiched between two Teflon plates and hot pressing at 60 °C under a constant presure of 5 MPa for 10 min. For a comprehensive compositional analysis, the weight ratio of Ti_3_C_2_T*_x_* to TOCNFs was regulated at 4:1, 3:2, 2:3, and 1:4, while the overall mass of each specimen was strictly fixed at 25 mg. Pure Ti_3_C_2_T*_x_* (M25) and pure TOCNFs (F25) films were identically processed as references. The resulting composites are designated as Mx@Fy, wherein x and y signify the absolute mass of MXene and TOCNFs, respectively.

### 2.5. Characterization

To elucidate the morphological and microstructural features of the fabricated films, field-emission scanning electron microscopy (FE-SEM, JSM-6700F, JEOL Ltd., Akishima, Japan) operating in SE mode and high-resolution transmission electron microscopy (HR-TEM, JEM-2100F, JEOL Ltd., Akishima, Japan) were utilized. The topographical profiles of the TOCNFs and Ti_3_C_2_T*ₓ* dispersions were mapped using an atomic force microscope (AFM, Agilent 5500, Agilent Technologies Inc., Santa Clara, CA, United States) in tapping mode. The surface elemental composition and chemical states were probed through X-ray photoelectron spectroscopy (XPS, ESCALAB 250 Xi, Thermo Fisher, Waltham, MA, United States). The static water contact angle was determined with a contact angle analyzer (Contact angle meter, JC2000C1, Shanghai Powereach Co., Ltd., Shanghai, China).

Before mechanical evaluation, the film samples were sectioned into rectangular strips (9.5 mm × 25 mm) and equilibrated in a controlled environment (18 °C, 25% relative humidity) for at least 6 h. Tensile testing was performed on a microcomputer-controlled electronic universal testing machine (Zwick Z005) equipped with a 100 N load cell, utilizing a crosshead speed of 0.5 mm min^−1^. To ensure statistical reliability, at least five independent specimens were tested per formulation, with the final results reported as mean values. Film thickness was determined directly from cross-sectional SEM micrographs. For electrical characterization, the sheet resistance of the strips was recorded using a four-point probe setup (RTS-9, Guangzhou, China), and the corresponding electrical conductivity was derived in accordance with Equation (1):

(1) $$\sigma =\frac{1}{S}\cdot \frac{1}{R/L}=\frac{L}{R\cdot w\cdot t}$$

where σ, R, L, S, w, and t represent the electrical conductivity (S cm^−1^), sheet resistance (Ω sq^−1^), length (cm), cross-sectional area (cm^2^), width (cm), and thickness (cm), respectively.

Electrochemical evaluations were conducted in a standard three-electrode configuration (CHI760E workstation, Shanghai Chenhua Instrument Co., Ltd., Shanghai, China) with 1 M H_2_SO_4_ as electrolyte. An Ag/AgCl electrode and a platinum sheet served as the reference and counter electrodes, respectively. The prepared MXene-based films were cut into 1 cm × 2 cm pieces and directly employed as binder-free working electrodes. For the symmetric device, two identical MXene-based films were used as both the positive and negative electrodes, a cellulose filter paper was used as the separator, and the entire system was assembled into a standard CR2032 coin cell. Both cyclic voltammetry (CV) and galvanostatic charge–discharge (GCD) measurements were recorded within a potential window of −0.2 to 0.3 V. Electrochemical impedance spectroscopy (EIS) data were collected over a frequency range from 100 kHz down to 0.01 Hz. CV measurements were conducted at various scan rates, while GCD tests were performed at different current densities.

The specific capacitance ($C,mF{cm}^{-2},Fg^{-1}$) in the three-electrode system was calculated from the GCD curves according to the following Equation (2):

(2) $$C_{s}^{\prime }=(I\cdot t)\cdot {\left(∆U\cdot S\right)}^{-1}$$

The variable $C_{s}^{\prime }$ denotes the areal capacitance of the working electrode, ∆U is the voltage window (V), S (cm^2^) refers to the geometric area of the electrode.

The formulas for calculating the energy density and power density of symmetric supercapacitors are as follows:

(3) $$E=1/2\cdot C_{s}\cdot {∆V}^{2}$$

(4) $$P=\frac{E}{∆t}$$

where E (μWh cm^−2^) and P (μW cm^−2^) denote the energy density and power density, C_s_ (mF cm^−2^) is the area-specific capacitance, ∆V (V) is the operating potential window, and ∆t (s) is the discharge time.

Kinetic characterization of the film electrode was conducted to clarify the electrochemical energy storage mechanism. To investigate the energy storage process, the CV data recorded at various scan rates were interpreted using the following Equations (5) and (6):

(5) $$i=i_{s}+i_{d}={av}^{b}$$

(6) logi=b logv+loga

The i denotes the current, while $i_{s}$ and $i_{d}$ represent the response current contributed by the surface capacitance and response current contributed by the diffusion-controlled embedded capacitance, respectively. v is the scan rate, a and b are parameters. When b ≈ 1, indicates the electrode material comes from the contribution of surface capacitance. When b = 0.5, the charge storage is primarily governed by the intercalation capacitance contributed by the intercalation and deintercalation of electrolyte ions.

Formula (5) can be converted into the following form:

(7) i(V)=k1v+k2v12

where $k_{1}$ and $k_{2}$ serve as parameters, $k_{1}v$ and k2v12 refer to the surface and embedded capacitance contributions.

The impedance characteristics of individual electrodes were evaluated in a standard three-electrode cell containing a liquid electrolyte. The impedance of the system is calculated using Equation (8).

(8) $$Z=R_{s}+\frac{1}{j\omega C_{edl}+\frac{1}{R_{ct}+W}}-\frac{j}{\omega C_{f}}$$

$R_{s}$, $R_{ct}$, and $R_{sf}$ denote the combined series resistance, charge-transfer resistance, and surface resistances, respectively. $C_{edl}$ and $C_{f}$ represent the constant phase element related to electrical-double-layer capacitance and constant phase element related to pseudocapacitance, respectively. While W is Warburg impedance.

Based on the impedance spectra, the real (C′) and imaginary (C″) parts of the capacitances are as follows:

(9) $$C^{\prime }(\omega )=\frac{-Z\prime \prime (\omega )}{\omega {\left|Z(\omega )\right|}^{2}}$$

(10) $$C\prime \prime (\omega )=\frac{-Z\prime (\omega )}{\omega {\left|Z(\omega )\right|}^{2}}$$

where C′(ω) is the real part of the capacitance C(ω). At the low-frequency limit, the value of C’ is equivalent to the cell capacitance determined through galvanostatic discharge. Z′ and Z″ represent the real and imaginary fractions of the impedance, respectively, with ∣Z∣ denoting the absolute impedance magnitude (Ω).

## 3. Results and Discussion

### 3.1. Morphology and Structure Characterization of MXene-Based Films

**Figure 2:**
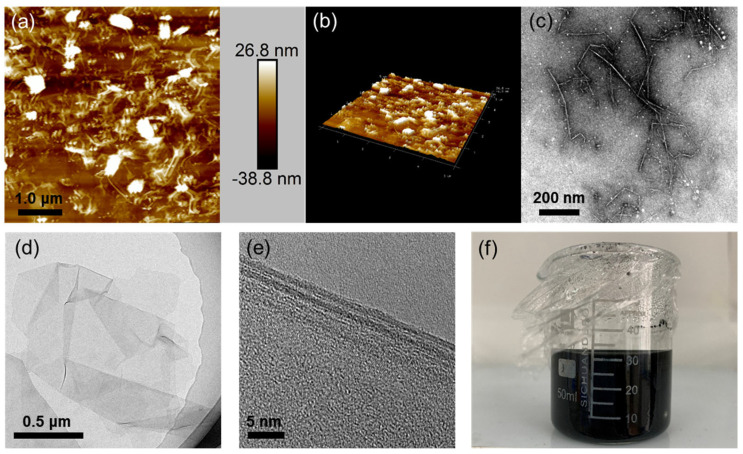
(**a**,**b**) AFM images of TOCNFs; (**c**) TEM image of TOCNFs; (**d**,**e**) TEM image of Ti_3_C_2_T*_x_* nanosheet; (**f**) Ti_3_C_2_T*_x_*/TOCNFs suspension.

The microscopic morphology of TOCNFs was characterized using AFM and TEM. As shown in Figure 2a–c, TOCNFs exhibit a highly entangled nanofibrillar network with an extremely high aspect ratio. Figure 2d,e presents the TEM images of Ti_3_C_2_T*_x_* nanosheets, revealing a large lateral dimension. Their high electron beam transparency confirms the successful exfoliation of the MAX phase into few-layer or single-layer MXene nanosheets. Figure 2f displays the Ti_3_C_2_T*_x_*/TOCNFs suspension, homogeneous, stable and dark ink-like, without any observable precipitation or agglomeration after standing. This excellent solution compatibility is attributed to the strong interfacial hydrogen bonding between the abundant oxygen-containing functional groups on the TOCNFs and the hydrophilic terminal groups of MXene [32].

**Figure 3:**
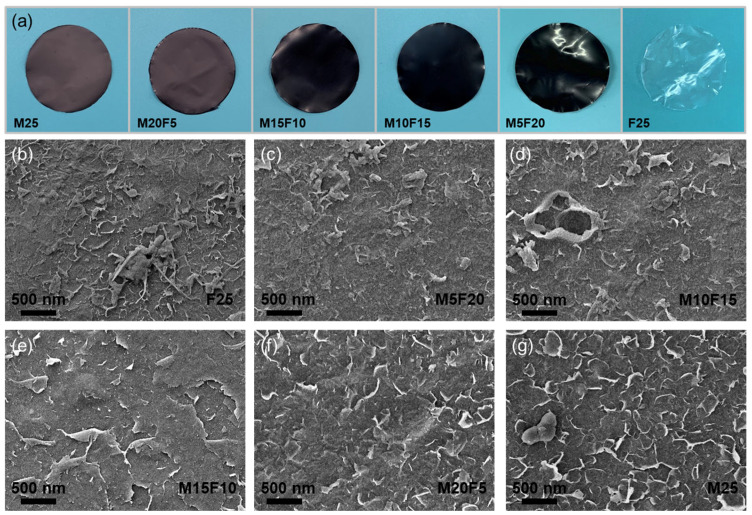
(**a**) MXene-based films with different mass ratios; (**b**–**g**) SEM images of the F25, M5@F20, M10@F15, M15@F10, M20@F5 and M25 films.

Figure 3a illustrates the macroscopic morphology of MXene-based films with varying mass ratios. The F25 film is transparent, as the MXene mass fraction increases, the color gradually transitions to the characteristic dark gray and metallic luster of the M25 film. All composite films exhibit smooth surfaces without obvious macroscopic cracks or structural defects. Figure 3b–g displays the microstructural evolution of the films. The F25 film (Figure 3b) presents a continuous, dense network. Upon the introduction of MXene (Figure 3c–f), the 2D MXene nanosheets are uniformly dispersed within the 1D TOCNFs without obvious agglomeration. Figure 3f shows the M20F5 composite film, where high-density MXene nanosheets overlap with a low content of TOCNFs to construct a dense, continuous 2D conductive network. This structure minimizes interfacial contact resistance, providing ultrafast electron transfer pathways for supercapacitors and establishing a highly efficient signal transduction network for flexible sensors [22]. Despite the high MXene mass fraction, the limited TOCNFs still function as an efficient nano-binder to eliminate interfacial defects. In contrast, the M25 film (Figure 3g) exhibits a typical stacked lamellar morphology, rendering it highly susceptible to internal stress concentration and intrinsic brittleness [33].Fabricated via hot-pressing densification, the MXene-based films feature TOCNFs wrapping and intercalating between the MXene layers. The synergistic application of pressure and heat drives the high-density hydrogen-bonding network of TOCNFs to tightly integrate with MXene, successfully constructing a nacre-inspired layered architecture. This structure suppresses MXene self-restacking, preserving abundant pores for electrolyte ion penetration and pseudocapacitive active sites.

### 3.2. Physicochemical Properties of MXene-Based Films

**Figure 4:**
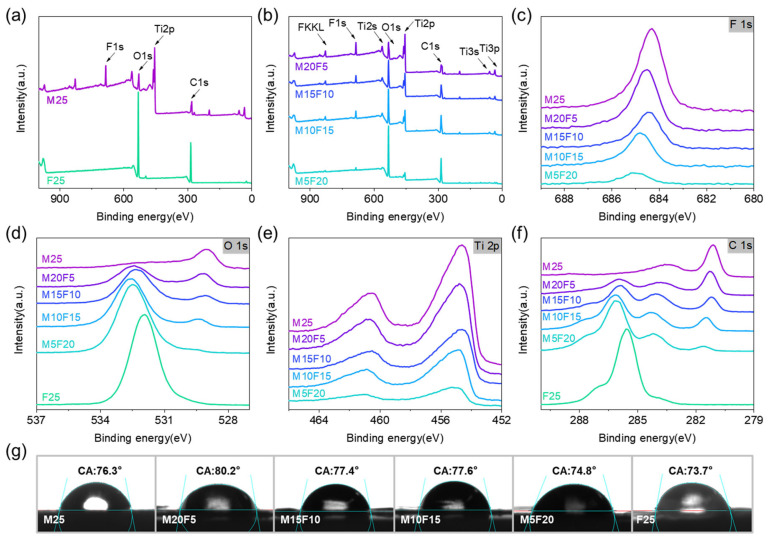
(**a**,**b**) XPS spectra of M25, M20F5, M15F10, M10F15, M5F20 and F25 films; (**c**–**f**) high-resolution XPS spectra of F 1s, O 1s, Ti 2p, and C 1s; (**g**) water contact angle images of Composite Films.

To deeply investigate the surface chemical composition and interfacial interactions between MXene and TOCNFs, XPS characterization was performed on the composite films. As shown in Figure 4a, originating from the abundant oxygen- and fluorine-containing terminal groups on the Ti_3_C_2_T*_x_* surface, the survey spectrum of M25 clearly exhibits the characteristic peaks of F 1s, O 1s, Ti 2p, and C 1s, whereas the survey spectrum of F25 only presents strong C 1s and O 1s characteristic peaks. Figure 4b displays the survey spectra of the composite films with different ratios, with the increasing proportion of TOCNFs, the peak intensities of Ti and F elements unique to MXene gradually weaken, indicating that the two materials achieved a uniform composite with tunable proportions. The high-resolution XPS spectra (Figure 4c–f) further reveal the chemical bonding states between MXene and TOCNFs. In the F 1s (Figure 4c) and Ti 2p (Figure 4e) spectra, the characteristic peak intensities weaken as the MXene content decreases. The stable peak profile of Ti 2p, together with the clear lattice fringes observed in TEM, collectively confirms that MXene maintained its intact phase structure and did not undergo excessive oxidation into TiO_2_ during the physical blending process. The C 1s spectrum in Figure 4f clearly shows the superposition of the C-Ti bonds of MXene and the C-C/C-O bonds of TOCNFs, confirming the physical blending of their carbon skeletons. Compared with the pure components, the binding energy of the O 1s peak (Figure 4d) in the composite films underwent a distinct shift; this chemical shift strongly proves that the abundant oxygen-containing functional groups on the TOCNFs surface formed a high-density intermolecular hydrogen-bonding network with the polar terminal groups on the MXene surface [22,23]. This dense hydrogen-bonding network effectively resolves the issue of weak interfacial adhesion between the 2D nanosheets, thereby significantly enhancing the mechanical properties of the composites.

The surface physicochemical properties of the films were evaluated via water contact angle tests (Figure 4g). The M25 film exhibits a contact angle of 76.3°, demonstrating intrinsic hydrophilicity. After incorporating a small amount of TOCNFs, the high-aspect-ratio TOCNFs intercalate between the MXene layers, leading to the remodeling of the microscopic morphology and roughness variation on the film surface, which causes the contact angle of M20F5 to slightly increase to 80.2°. With the increasing proportion of TOCNFs carrying abundant hydrophilic hydroxyl groups, the contact angle of the composite films gradually decreases to 73.7° for the F25 film. The moderate variations in surface hydrophilicity and wettability are beneficial for the composite films to maintain excellent ion accessibility in aqueous electrolytes, and they also improve the interfacial compatibility of the electrode materials, thereby guaranteeing the structural stability and electrochemical cycling lifespan of the device under high-rate charge–discharge conditions [34].

### 3.3. Electrochemical Performance of MXene-Based Films

**Figure 5:**
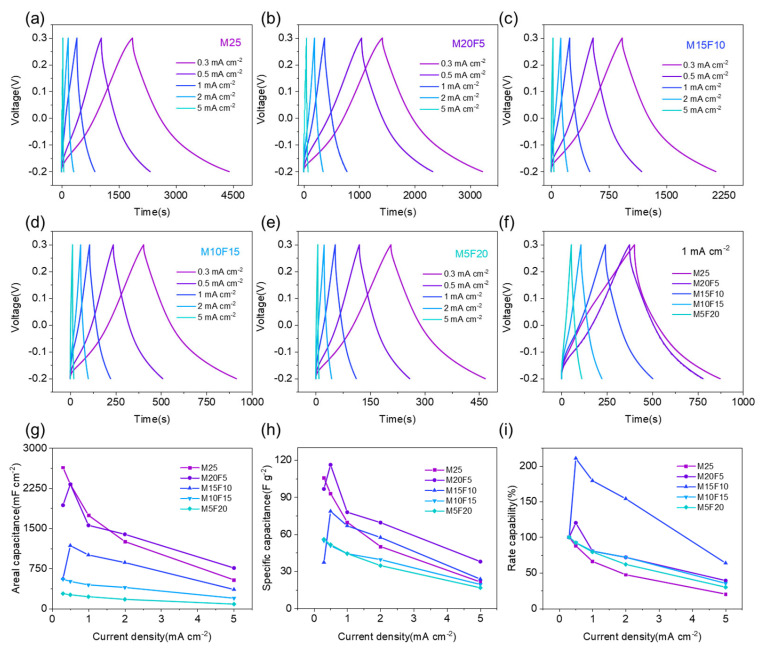
(**a**–**e**) GCD curves of M25, M20F5, M15F10, M10F15, M5F20 films at different current densities; (**f**) GCD profiles at 1 mA cm^−2^; (**g**,**h**) areal and specific capacitance of M25, M20F5, M15F10, M10F15, M5F20 films; (**i**) rate capability of M25, M20F5, M15F10, M10F15, M5F20 films based on GCD profiles.

Figure 5a–e illustrates the GCD curves of the composite films with various blending ratios at current densities ranging from 0.3 to 5 mA cm^−2^. All obtained GCD curves exhibit highly symmetric, nearly isosceles triangular shapes without distinct charge–discharge voltage plateaus. The energy storage mechanism of the composite films is co-dominated by the electrical double-layer capacitance and the Faradaic pseudocapacitance originating from the abundant hydrophilic terminal groups on the MXene surface. Consequently, the GCD profiles display a slight deviation from an ideal triangular shape. At the moment of charge–discharge polarity reversal, the curves present a negligible voltage drop, demonstrating the excellent intrinsic electrical conductivity and ultralow equivalent series resistance of the electrode materials [35,36]. Figure 5f compares the GCD profiles of all samples at a specific current density of 1 mA cm^−2^. Both M25 and M20F5 films exhibit remarkably prolonged discharge times, signifying their superior absolute charge storage capacities.

The evolution trends of the areal capacitance and specific capacitance, derived from the GCD curves, are presented in Figure 5g,h and Table 1 and Table 2. At a low current density of 0.3 mA cm^−2^, the M25 film, benefiting from its maximum loading of electrochemically active materials, delivers the highest areal capacitance of 2637.66 mF cm^−2^ and a specific capacitance of 105.51 F g^−1^. As the current density increases, electrolyte ions fail to diffuse promptly into the deep microporous network of the materials, confining charge storage predominantly to the outer electrode surface. This results in varying degrees of capacitance decay across all films. This phenomenon is particularly pronounced in the pure M25 film, restricted by strong van der Waals forces and severe self-restacking, the hindered deep penetration of the electrolyte leads to a drastic capacity degradation [37,38,39]. When the current density exceeds 1.5 mA cm^−2^ up to 5 mA cm^−2^, the areal capacitance of the M20F5 composite film surpasses that of the M25 film, outperforming all other samples. Anomalously, both the areal and specific capacitances of the M20F5 and M15F10 samples at 0.5 mA cm^−2^ are higher than their initial values at 0.3 mA cm^−2^. This indicates that the abundant hydrophilic functional groups on the TOCNFs surface facilitate the deep infiltration of the electrolyte, thereby achieving the electrochemical activation of deeply hidden active sites.

The rate capability test results are summarized in Figure 5i and Table 3. Using the capacitance value at 0.3 mA cm^−2^ as the baseline, when the current density increases to 5 mA cm^−2^, the capacitance retention of the M25 film plummets to 20.33% due to the severe self-restacking effect. In contrast, the M20F5 composite film maintains a substantially higher capacitance retention of 39.31% under the same harsh conditions. Although the M15F10 sample with a higher TOCNFs content exhibits a numerically higher rate retention percentage, its absolute capacitance value (Figure 5f) is significantly lower, thereby lacking practical application viability. The exceptional electrochemical performance of the M20F5 composite film is attributed to the effective intercalation of 1D, high-aspect-ratio TOCNFs between the 2D MXene nanosheets, successfully constructing a nacre-inspired microscopic heterostructure [40]. Without disrupting the intrinsic continuous electron transport network of MXene, this architecture remodels the microscopic pore structure of the film and significantly improves the ion transport efficiency of the composite. Furthermore, the dense interfacial hydrogen-bonding network formed between TOCNFs and the terminal groups of MXene provide hopping conduction channels for hydrogen ions. This enables ultrafast proton transport, ultimately allowing the M20F5 film to maintain an outstanding capacity retention rate even at high current densities.

**Figure 6:**
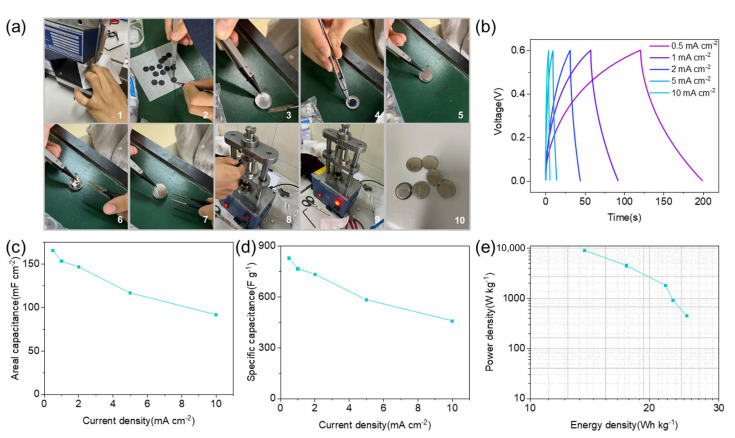
(**a**) Encapsulate M20F5 composite films for coin cell assembly; (**b**–**d**) GCD curves, areal capacitance and specific capacitance of the assembled coin cell based on the M20F5 composite films; (**e**) Ragone plot displaying the energy and power densities of the M20F5-based coin cell.

The M20F5 composite film was assembled into CR2032 coin cell for electrochemical evaluation (Figure 6a). Figure 6b displays the GCD curves of the device at varying current densities from 0.5 to 10 mA cm^−2^. The curves exhibit a nearly symmetric triangular shape with no discernible voltage drop at the polarity reversal point. This demonstrates the device’s excellent capacitive energy storage capability, low equivalent series resistance, and reversible electrochemical kinetics, indicating the successful construction of highly efficient electron conduction network within the device. The detailed electrochemical performance of the assembled coin cell is summarized in Table 4. At a current density of 0.5 mA cm^−2^, the M20F5 composite film delivers an outstanding areal capacitance of 165.78 mF cm^−2^ and a specific capacitance of 828.92 F g^−1^, with a prolonged discharge time of 198.94 s. At such a low rate, electrolyte ions can fully penetrate and access the electrochemical active sites within the electrode material. When the current density increased to 10 mA cm^−2^, the device still retains an areal capacitance of 91.67 mF cm^−2^ and a specific capacitance of 458.33 F g^−1^, corresponding to a capacitance retention of approximately 55.29%.

Figure 6e and Table 4 further illustrate the exceptional energy and power densities of the device. At a current density of 0.5 mA cm^−2^, the device achieves an energy density of 24.87 Wh kg^−1^ at a power density of 450.45 W kg^−1^. Upon increasing the current density to 10 mA cm^−2^, the power density surges to 9500 W kg^−1^. Although the energy density decreases, it remains at a commendable 13.75 Wh kg^−1^, demonstrating significant potential for practical applications. The introduction of TOCNFs with high aspect ratios as physical spacers successfully constructs a nacre-inspired lamellar network and a dense hydrogen-bonding network. This structural design effectively alleviates the self-restacking of MXene nanosheets to expose more pseudocapacitive active sites, thereby optimizing the microscopic networks for rapid ion transport during charge or discharge processes, thereby maintaining the structural stability of the device under high-current-density operations [41,42,43].

### 3.4. Applications of the MXene-Based Film Pressure Sensor

**Figure 7:**
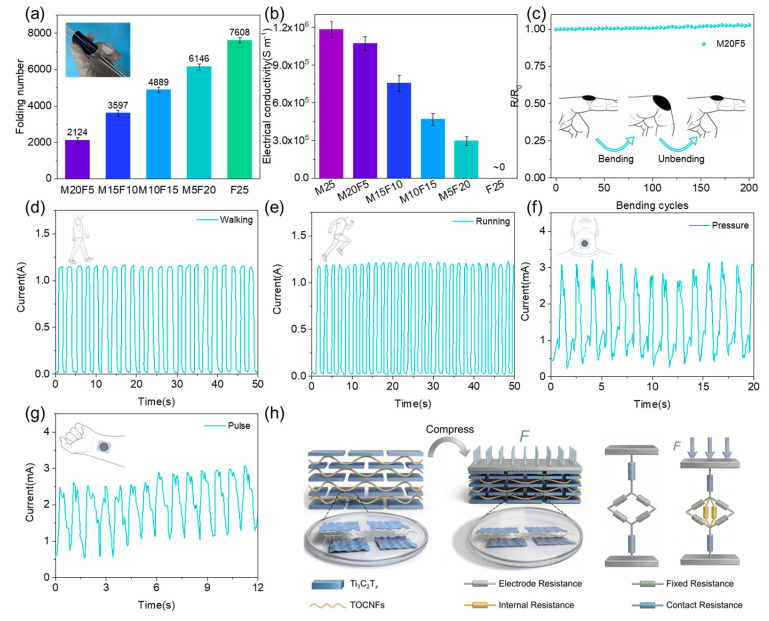
(**a**) Folding test; (**b**) conductivity measurement results; (**c**) relative resistance with the number of bending cycles; (**d**,**e**) current response of the signal when walking or running; (**f**) current response for speaking the word “pressure”; (**g**) current response of the pulse at the wrist; (**h**) sensing mechanism.

The mechanical flexibility and electrical stability of the MXene-based films were systematically characterized (Figure 7a–c). As shown in Figure 7a, the M20F5 composite film can tightly adhere to a small-radius glass tube without any visible cracking, delamination, or peeling, demonstrating that the introduction of 1D TOCNFs significantly enhances the mechanical robustness, macroscopic flexibility, and surface conformability of the film. The folding endurance of the films increases linearly with the higher TOCNFs content, effectively overcoming the intrinsic brittleness of pure MXene films. Although the addition of insulating TOCNFs as physical spacers inevitably compromises the overall electrical conductivity (Figure 7b), the M20F5 composite film achieves an optimal electromechanical balance, maintaining an ultrahigh conductivity of 1.07 × 10^6^ S m^−1^ (90.68% of the M25 film), while enduring approximately 2124 folding cycles. Furthermore, the M20F5 film exhibits remarkable dynamic mechanical fatigue resistance, with its relative resistance curve remaining highly stable over 200 continuous bending cycles (Figure 7c). The exceptional electrical retention suggests that the internal conductive pathways do not suffer from irreversible disconnection. Microscopically, as schematically proposed in Figure 7h, the folding-unfolding process triggers a highly reversible sliding-and-recovery behavior among the constituent layers. When subjected to bending strain, the 1D high-aspect-ratio TOCNFs serve as elastic nano-binders that permit localized, controllable relative sliding of 2D MXene sheets to delocalize stress concentrations. Upon unloading, the high-density interfacial hydrogen-bonding network acts as a molecular spring, driving the slipped flakes back to re-establish tight contact area and contact resistance. Consequently, the microstructural integrity is well preserved against internal crack propagation, ensuring outstanding long-term operational reliability.

The M20F5 piezoresistive sensor was further applied for real-time human motion and physiological signal monitoring. It successfully captured the distinct differences in waveform characteristics of the current amplitude and response frequency during walking and running (Figure 7d,e), proving its potential for bimodal gait recognition and physical activity tracking. Beyond large-scale motions, the sensor also exhibits high sensitivity to subtle physiological vibrations. When attached to the throat (Figure 7f), the sensor precisely records the micro-vibrations of the vocal cords during the pronunciation of the word “pressure,” yielding highly repeatable and characteristic current pulse waveforms. When mounted on the wrist (Figure 7g), it accurately tracks the human radial pulse with clear and periodic responses. Figure 7h schematically illustrates the working mechanism of the piezoresistive sensor. Upon the application of pressure, the internal resistance is connected in parallel with the fixed resistance, and in series with the contact resistance and electrode resistance, thereby reducing the total resistance of the sensor. When the pressure is released, the sensor gradually recovers to its initial state.

## 4. Conclusions

In summary, a facile vacuum filtration and hot-pressing densification strategy was developed to construct MXene-based films with a nacre-inspired lamellar structure. The formulated M20F5 composite film achieved a coordinated balance between mechanical robustness and electrical performance, exhibiting stable dynamic fatigue resistance by maintaining an electrical conductivity of 1.07 × 10^6^ S m^−1^ after enduring 2124 folding cycles. For energy storage, the open lamellar network with suppressed self-restacking exposes abundant pseudocapacitive active sites. The assembled symmetric supercapacitor delivers a specific capacitance of 828.92 F g^−1^ at 0.5 mA cm^−2^ and maintains an energy density of 13.75 Wh kg^−1^ at a power density of 9500 W kg^−1^. Operating as a piezoresistive sensor driven by the reversible sliding and contact resistance modulation of microscopic layers under pressure, the film enables the distinction of motions spanning from macroscopic bimodal gait recognition to subtle physiological signals. By integrating reproducible mechanical durability, energy storage, and piezoresistive sensing capabilities, these MXene-based films offer a viable structural strategy for next-generation supercapacitors and flexible wearable devices.

## Figures and Tables

**Table 1:** Areal capacitance of the composite films at different current densities.

Current Density (mA cm^−2^)	M25 (mF cm^−2^)	M20F5 (mF cm^−2^)	M15F10 (mF cm^−2^)	M10F15 (mF cm^−2^)	M5F20 (mF cm^−2^)
0.3	2637.66	1932.66	559.26	547.98	279.7116
0.5	2323.1	2323.1	1177.83	508.3	258.23
1	1744.8	1557	1002.8	442.8	222.068
2	1252.96	1388.92	861.12	395.6	173.46
5	536.25	758.73	356.73	194.2	83.937

**Table 2:** Specific capacitance of the composite films at different current densities.

Current Density (mA cm^−2^)	M25 (F g^−2^)	M20F5 (F g^−2^)	M15F10 (F g^−2^)	M10F15 (F g^−2^)	M5F20 (F g^−2^)
0.3	105.51	96.63	37.28	54.8	55.94
0.5	92.92	116.16	78.52	50.83	51.65
1	69.79	77.85	66.85	44.28	44.41
2	50.12	69.45	57.41	39.56	34.69
5	21.45	37.94	23.78	19.42	16.79

**Table 3:** Rate capability of the composite films at different current densities.

Current Density (mA cm^−2^)	M25 (%)	M20F5 (%)	M15F10 (%)	M10F15 (%)	M5F20 (%)
0.3	100	100	100	100	100
0.5	88.08	120.21	210.62	92.54	92.33
1	66.15	80.57	179.29	80.61	79.39
2	47.5	71.93	154	71.97	62.01
5	20.33	39.31	63.79	35.43	29.99

**Table 4:** Electrochemical performance of the assembled coin cell at different current densities.

Current Density (mA cm^−2^)	Discharge Time (s)	Areal Capacitance (mF cm^−2^)	Specific Capacitance (F g^−1^)	Energy Density (Wh kg^−1^)	Power Density (W kg^−1^)
0.5	198.94	165.78	828.92	24.87	450.45
1	91.98	153.30	766.50	22.99	902.39
2	44.02	146.73	733.67	22.01	1799.91
5	14.00	116.67	583.33	17.50	4500
10	5.50	91.67	458.33	13.75	9500

## Data Availability

The data presented in this study are available on request from the corresponding author.

## Abbreviations

The following abbreviations are used in this manuscript:

1D	One-Dimensional
2D	Two-Dimensional
TEMPO	2,2,6,6-Tetramethylpiperidine-1-oxyl
TOCNFs	TEMPO-oxidized Cellulose Nanofibrils
MXene	2D Transition Metal Carbides and Nitrides
MAX	Ternary Layered Transition Metal Carbides/Nitrides (MAX phase)
Ti_3_C_2_T*_x_*	2D Titanium Carbide MXene
DI	Deionized Water
AFM	Atomic Force Microscope
HR-TEM	High-Resolution Transmission Electron Microscopy
FE-SEM	Field-Emission Scanning Electron Microscopy
XPS	X-ray Photoelectron Spectroscopy
EIS	Electrochemical Impedance Spectroscopy
CV	Cyclic Voltammetry
GCD	Galvanostatic Charge-Discharge
